# CiCXCL14 Exhibits Broad-Spectrum Bactericidal Activity and Protects Grass Carp Against *Aeromonas hydrophila* Infection

**DOI:** 10.3390/ani16162458

**Published:** 2026-08-07

**Authors:** Ya-Zhen Hu, Yu Chen, Weicheng Wang, Bo Yuan, Youfeng Xie, He Zhao, Huijie Chen, Wentao Zhu

**Affiliations:** 1 State Key Laboratory for Quality and Safety of Agro-Products, Ningbo University, Ningbo 315211, China; 2 College of Life Science and Technology, Tarim University, Alar 843300, China; 3 International Joint Research Center for Coral Reef Ecology of Hainan Province, School of Ecology, Hainan University, Haikou 570228, China

**Keywords:** CXCL14, antimicrobial protein, DNA binding, membrane disruption, grass carp, *Aeromonas hydrophila*

## Abstract

Chemokines are best known for orchestrating leukocyte migration; however, accumulating evidence indicates that mammalian CXCL14 also functions as a direct antimicrobial effector of innate immunity. Nevertheless, the molecular basis underlying the bactericidal activity of fish chemokines remains largely unexplored. In this study, we identified and functionally characterized *Ctenopharyngodon idella* CXCL14 (CiCXCL14) as a broad-spectrum antimicrobial protein. CiCXCL14 is predominantly expressed in immune-related organs and is significantly upregulated upon *Aeromonas hydrophila* challenge, suggesting an active role in antibacterial defense. Recombinant CiCXCL14 exhibited potent, concentration-dependent bactericidal activity against *E. coli*, *A. hydrophila*, *P. fluorescens*, *A. veronii*, *S. aureus*, and *S. agalactiae*. Time-kill assays further demonstrated that CiCXCL14 exhibited rapid bactericidal activity, achieving near-complete killing of *S. aureus* within 40 min and *E. coli* within 160 min. Mechanistically, CiCXCL14 disrupts bacterial membrane integrity and interacts with purified bacterial genomic DNA, indicating that membrane damage and DNA interaction may both contribute to its bactericidal activity. Notably, CiCXCL14 retained substantial antibacterial activity after heat treatment, whereas its activity gradually decreased with increasing NaCl concentrations. In vivo, administration of recombinant CiCXCL14 improved survival rates in grass carp challenged with a lethal dose of *A. hydrophila* and reduced bacterial burdens in the liver, spleen, and trunk kidney. Collectively, these findings identify CiCXCL14 as a teleost chemokine with direct antibacterial activity and demonstrate that it contributes to antibacterial defense through membrane disruption and interaction with bacterial DNA. These findings provide a foundation for future studies investigating the potential application of CiCXCL14 in aquaculture disease control.

## Simple Summary

Bacterial diseases are a major challenge in aquaculture, creating an urgent need for new disease-control strategies. In this study, we investigated the role of a natural immune protein, CXCL14, from grass carp in protecting fish against bacterial infections. We found that this protein was highly expressed in immune-related tissues and was upregulated after bacterial challenge. Recombinant CXCL14 showed broad-spectrum antibacterial activity against several important fish pathogens and protected grass carp by reducing bacterial burdens and improving survival after infection. Laboratory experiments also showed that the protein disrupted bacterial membranes and interacted with purified bacterial DNA in vitro. These findings improve our understanding of the antibacterial function of fish CXCL14 and provide a basis for future studies on its potential application in aquaculture.

## 1. Introduction

Chemokines are a superfamily of small secreted cytokines that primarily regulate leukocyte migration and immune cell trafficking [1,2]. Based on the arrangement of conserved cysteine residues, chemokines are classified into the CXC, CC, CX3C and XC subfamilies [1,2,3]. Besides their canonical chemotactic functions, increasing evidence indicates that several chemokines possess direct antimicrobial activities and function as effector molecules of innate immunity [1,2,4,5,6]. Similar to classical antimicrobial proteins (AMPs), antimicrobial chemokines can directly target invading microorganisms through interactions with bacterial membranes and intracellular targets [1,2,6]. To date, 23 out of 46 human chemokines have been shown to exhibit such activity [4]. This antimicrobial function operates independently of chemokine receptor engagement, positioning chemokines as a unique class of dual-function molecules that bridge the chemotactic network and the classical AMP arsenal of innate immunity [2,4].

In teleost fish, the antimicrobial function of chemokines has gained increasing recognition over the past decade [7]. The first evidence came from the characterization of a rainbow trout CC chemokine, CK11, which was shown to exert potent antimicrobial activity against a wide range of Gram-positive and Gram-negative fish pathogens [8]. Since then, direct bactericidal activity has been documented in multiple fish chemokines. For example, grass carp CXCL20a/b (CiCXCL20a/b) exhibits broad-spectrum bactericidal activity by targeting bacterial membrane lipids, causing membrane perforation and cytoplasmic leakage [5,6]. Similarly, Japanese flounder CXCL8 and CXCL10 have been reported to possess direct bactericidal effects [9,10]. Collectively, these studies demonstrate that direct antimicrobial activity is a common functional feature of teleost chemokines, highlighting their important roles in innate immune defense against bacterial pathogens.

Among chemokines endowed with antimicrobial activity, CXCL14 has attracted particular attention due to its distinctive biological properties. In mammals, CXCL14 is a homeostatic chemokine with an atypical yet highly conserved primary structure, characterized by a short N-terminal region and high sequence identity between human and mouse orthologs [11,12]. It is constitutively and abundantly expressed in epithelial tissues, including the skin and respiratory tract [11,12]. Early studies demonstrated that human CXCL14 exhibits potent and broad-spectrum antimicrobial activity against cutaneous Gram-positive bacteria, *Candida albicans*, and *Escherichia coli* [12]. Subsequent studies revealed that CXCL14 kills respiratory pathogens such as *Pseudomonas aeruginosa*, *Streptococcus mitis* and *Streptococcus pneumoniae* in a dose-dependent manner, partially through membrane depolarization and rupture [11]. CXCL14-deficient mice exhibited impaired clearance of *S. pneumoniae* from the lungs in vivo, underscoring the physiological relevance of this chemokine in host defense [11]. Structural analyses have revealed that CXCL14 contains highly cationic regions and amphipathic motifs resembling those found in classical AMPs, suggesting that direct antimicrobial activity may represent an evolutionarily conserved function of this chemokine. Moreover, mammalian CXCL14 has been reported to directly interact with bacterial DNA, further supporting its potential as a multifunctional antimicrobial effector [13,14].

Teleost fish inhabit microorganism-rich aquatic environments and rely heavily on innate immune mechanisms to combat pathogen invasion. Numerous AMPs have been identified in teleosts and shown to exert antimicrobial effects through membrane disruption, intracellular targeting, and immunomodulation [15,16,17]. Grass carp (*Ctenopharyngodon idella*) is one of the most extensively cultured freshwater fish species, yet it is highly susceptible to a variety of bacterial pathogens, including *Aeromonas hydrophila*, *Aeromonas veronii* and *Flavobacterium columnare* [18,19,20,21]. However, despite accumulating evidence that several teleost chemokines possess direct antimicrobial activity, the biological function of fish CXCL14 remains enigmatic. To date, studies on teleost CXCL14 have largely focused on its expression patterns and transcriptional responses to immune stimulation, whereas its direct antimicrobial activity, bactericidal mechanism, and in vivo protective function have not been experimentally characterized. For example, transcriptomic analyses have identified CXCL14 as a constitutively expressed chemokine in catfish, suggesting a role in immune homeostasis [22]. Therefore, the antimicrobial function and mechanism of fish CXCL14 remain to be elucidated.

Although antimicrobial functions have been reported for several chemokines in vertebrates, the molecular mechanisms underlying the bactericidal activity of fish CXCL14, particularly whether it acts through membrane disruption, DNA binding, or both, remain largely unexplored. Moreover, the in vivo protective efficacy of fish CXCL14 against bacterial infections has not been experimentally demonstrated. In the present study, we aimed to identify and functionally characterize CiCXCL14, with a particular focus on evaluating its direct bactericidal activity and investigating its underlying antibacterial mechanisms. We further assessed its in vivo protective role against bacterial infection in grass carp. This study provides new insights into the antibacterial function of teleost CXCL14 and advances our understanding of the roles of chemokines in fish innate immunity.

## 2. Materials and Methods

### 2.1. Ethics Statement

Grass carp weighing 20 ± 5 g were obtained from the Baicheng Grass Carp Breeding Base (Aksu Prefecture, China). Fish were acclimated for two weeks in aerated freshwater tanks maintained at 28 ± 1 °C under a 12 h light/12 h dark photoperiod and fed a commercial diet twice daily. Prior to bacterial challenge, fish were fasted for 24 h and randomly assigned to the experimental groups. All animal experiments were conducted in accordance with the guidelines for the care and use of laboratory animals and were approved by the Ethical Committee on Animal Research at Tarim University (Approval No. 20260310009; Approval Date: 1 March 2026). Fish were anesthetized with MS-222 (100 mg/L) before injection and tissue sampling. Fish reaching predefined humane endpoints were immediately euthanized with an overdose of MS-222 (300 mg/L). Every effort was made to minimize animal suffering throughout the study.

### 2.2. Sequence Analysis, Domain Identification and Phylogenetic Analysis of CXCL14

**Figure 1:**
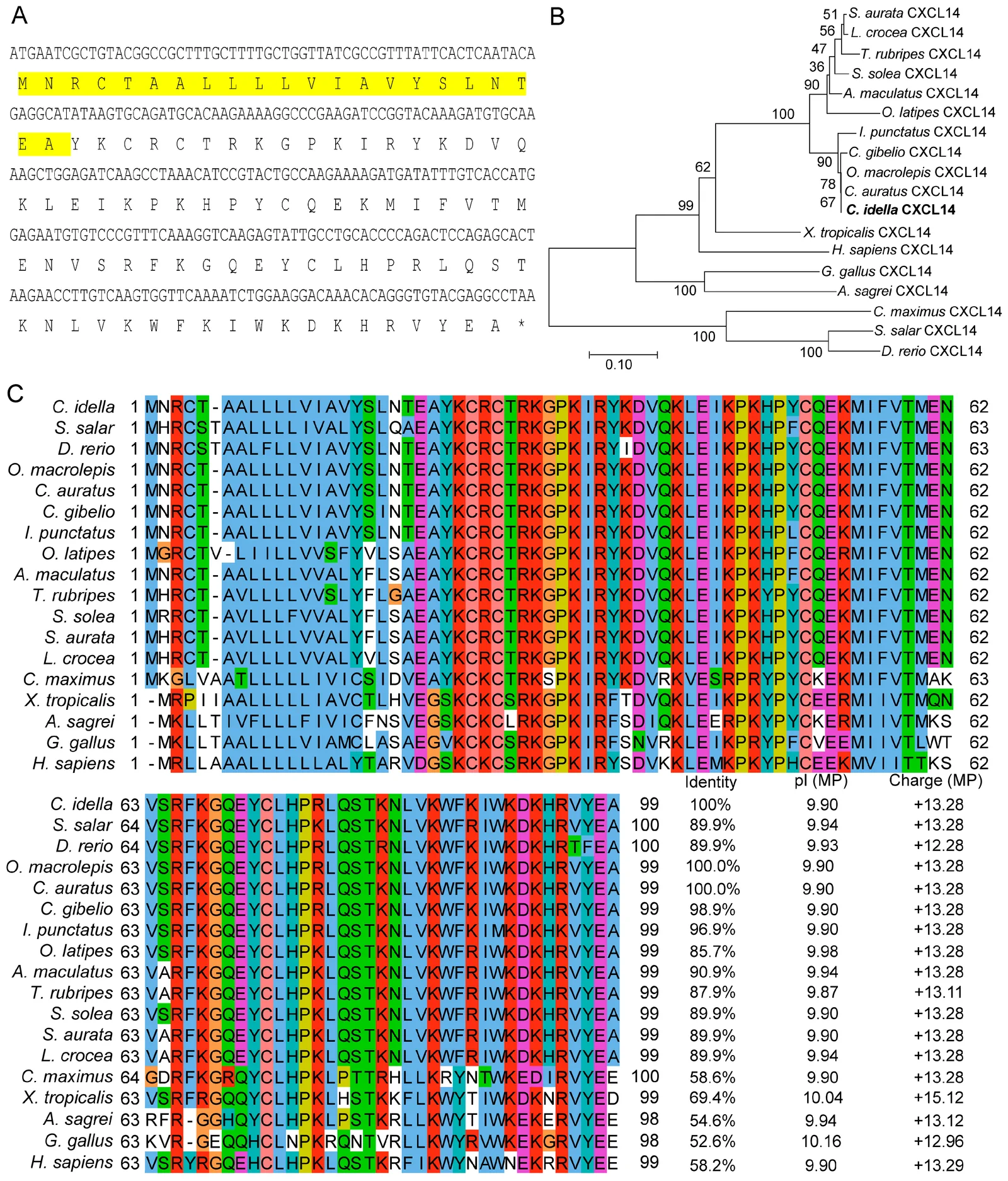
Sequence analysis, structural prediction, and evolutionary conservation of CXCL14. (**A**) The coding sequence and the corresponding translated protein of grass carp CXCL14. The N-terminal signal peptide of grass carp CXCL14 is marked by a yellow box. (**B**) A neighbor-joining phylogenetic tree constructed using CXCL14 amino acid sequences from grass carp and selected vertebrate species. Bootstrap values (based on 1000 replicates) are indicated at the main bifurcation points. (**C**) Comparative alignment of CXCL14 protein sequences from grass carp and multiple organisms. Fully conserved residues are indicated by colored shading. The right panel shows the percentage identity, calculated pI values, and estimated molecular net charges for each aligned sequence, with the CiCXCL14 sequence set as the reference for identity calculations. *H. sapiens* CXCL14 accession number: NP_004878.3, *G. gallus* CXCL14 accession number: NP_990043.1, *A. sagrei* CXCL14 accession number: XP_060621295.1, *X. tropicalis* CXCL14 accession number: NP_001107714.1, *I. punctatus* CXCL14 accession number: NP_001187133.1, *S. salar* CXCL14 accession number: NP_001134297.1, *T. rubripes* CXCL14 accession number: XP_011609242.2, *S. solea* CXCL14 accession number: XP_058506465.1, *O. macrolepis* CXCL14 accession number: XP 058653065.1, *C. gibelio* CXCL14 accession number: XP_052431282.1, *A. maculatus* CXCL14 accession number: KAN4269588.1, *S. aurata* CXCL14 accession number: XP_030252468.1, *O. latipes* CXCL14 accession number: XP_004073058.1, *L. crocea* CXCL14 accession number: XP_010741229.1, *C. auratus* CXCL14 accession number: XP_026081151.1, *D. rerio* CXCL14 accession number: NP_571702.1, *C. idella* CXCL14 accession number: XP_051716659.1, *C. maximus* CXCL14 accession number: XP_078407048.1.

The amino acid sequences of CXCL14 homologs from representative vertebrate species were retrieved from the NCBI database (https://www.ncbi.nlm.nih.gov) using online website of BLASTp (https://blast.ncbi.nlm.nih.gov/Blast.cgi?PAGE=Proteins, accessed on 5 August 2026) searches with the grass carp CXCL14 sequence as the query. As shown in Figure 1, taxon selection covered major vertebrate lineages, including teleosts (Cypriniformes, Perciformes, Salmoniformes, Siluriformes), tetrapods (amphibians, reptiles, birds, mammals), and cartilaginous fish as an outgroup. When multiple isoforms were available, the best-annotated RefSeq sequence was selected. The accession numbers for all sequences used in this analysis are provided in the Figure 1 caption. Multiple alignments of the protein sequences were generated using the ClustalW program within Jalview. The phylogenetic tree was constructed using MEGA 7 based on the neighbor-joining method with p-distance. The reliability of each node was estimated by bootstrapping with 1000 replicates. The tree was rooted using CXCL14 sequences from cartilaginous fish (e.g., Chiloscyllium plagiosum/Callorhinchus milii) as the outgroup. A scale bar indicating substitutions per site was included in the tree. The theoretical isoelectric point (pI) and net charge of the mature CXCL14 proteins included in the phylogenetic analysis were calculated using the ProtParam tool available on the DNASTAR software 7.1. Net charge was calculated from the amino acid sequence at neutral pH (pH 7.0). The predicted three-dimensional structure of grass carp CXCL14 was generated using AlphaFold3 (https://alphafold.ebi.ac.uk/). Model confidence was assessed using pLDDT (predicted Local Distance Difference Test) and PAE (Predicted Aligned Error). The mature CiCXCL14 model showed that ipTM (interface predicted template modeling score) = pTM (predicted template modeling score) = 0.68, with the core chemokine fold (residues 19–37 and 50–68) of pLDDT scoring above 90, indicating high confidence; the N-terminal region (residues 2–18) and the connecting loop region (residues 38–49) showed pLDDT scores between 70 and 90, indicating relatively greater structural flexibility. The predicted aligned error (PAE) matrix was uniformly low across almost the entire protein, indicating high confidence in the relative positioning of residues within the CXCL14 structure. Slightly increased PAE values were only observed at the N- and C-terminal regions, consistent with their intrinsic flexibility. Structural superimposition of CiCXCL14 and HsCXCL14 was performed using the align command in PyMOL v2.5 based on Cα atoms. The resulting RMSD was calculated in ångströms (Å) and visualized using PyMOL.

### 2.3. Expression and Purification of Recombinant CiCXCL14 Protein

The coding sequence corresponding to the mature protein region of CiCXCL14 (residues 23–99, corresponding to a 77-amino-acid mature peptide) was amplified. The coding sequence was amplified using the following primers: forward, 5′-ATCGCTCGAGATGCATCATCATCATCATCACTATAAGTGCAGATGCACAAGAAAAG-3′, and reverse, 5′-ATCGGGATCCTTAGGCCTCGTACACCCTGTGTT-3′, containing *Xho*I and *Bam*HI restriction sites, respectively. The mature CiCXCL14 sequence was cloned into the pET-15a expression vector, which introduced a 6×His tag for purification. The predicted molecular weight of mature CiCXCL14 and the His-tagged recombinant protein was approximately 10 kDa and 11 kDa, respectively. The recombinant plasmid was transformed into *E. coli* BL21 (DE3) competent cells, and protein expression was induced with 0.5 mM IPTG when the culture reached an OD600 of 0.6–0.8. After induction at 37 °C for 5 h, bacterial cells were harvested and lysed by sonication. The recombinant His-tagged CiCXCL14 protein was purified using Ni-NTA affinity chromatography according to the manufacturer’s instructions and subsequently dialyzed against PBS. Protein purity was evaluated by densitometric analysis of Coomassie Brilliant Blue-stained SDS-PAGE gels under reducing conditions using ImageJ software 1.52a (National Institutes of Health, USA), and the protein concentration was determined using a bicinchoninic acid (BCA) assay kit. The purified recombinant protein was subjected to endotoxin removal with an Endotoxin Removal Kit (Smart-Lifesciences, Changzhou, China) according to the manufacturer’s instructions. Residual endotoxin levels were measured using EC Endotoxin Test Kits (Bioendo, Xiamen, China) following the manufacturer’s protocol and were confirmed to be below 0.1 EU/μg protein. The recombinant protein used in all subsequent functional assays corresponded to the mature form of CiCXCL14. In addition, an unrelated recombinant protein, thioredoxin A (TrxA), was expressed from the pET-32a vector, purified using the same protocol as recombinant CiCXCL14, and included as a negative protein control where indicated.

### 2.4. Bacterial Challenge for Tissue Expression Analysis

To evaluate the expression dynamics of CiCXCL14 in response to bacterial infection, healthy grass carp (20 ± 5 g) were intraperitoneally injected with 100 μL of sterile PBS containing *A. hydrophila* at a dose of 1 × 10^6^ CFU per fish. At 0 h (pre-injection), 12 h, 24 h, and 48 h post-injection, 6 fish were randomly sampled at each time point. The liver, spleen, head kidney, gill, and skin were aseptically collected, immediately frozen in liquid nitrogen, and stored at −80 °C until RNA extraction. The expression levels of CiCXCL14 in these tissues were then determined by qPCR as described above.

### 2.5. Quantitative Real-Time PCR (qPCR) Analysis

Total RNA was extracted from various tissues using TRIzol reagent (Invitrogen, Carlsbad, CA, USA) according to the manufacturer’s instructions. RNA quality and concentration were assessed by agarose gel electrophoresis and spectrophotometry (NanoDrop 2000, Waltham, MA, USA). First-strand cDNA was synthesized from 1 µg of total RNA using a HiScript IV All-in-One Ultra RT SuperMix for qPCR (Vazyme, Nanjing, China) following the manufacturer’s protocol.

qPCR was performed using TB Green^®^ Premix Ex Taq™ II (Tli RNaseH Plus) (Takara, Kyoto, Japan) on a LightCycler^®^ 96 System (Roche, Basel, Switzerland). The thermal cycling conditions were as follows: initial denaturation at 95 °C for 30 s, followed by 40 cycles of 95 °C for 5 s and 60 °C for 30 s, with a final melting curve analysis step (60 and 95 °C) to confirm amplification specificity. The primer sequences used for amplification were as follows: CiCXCL14 F: GCACAAGAAAAGGCCCGAAG, CiCXCL14 R: GGGGTGCAGGCAATACTCTT; 18S rRNA (used as the internal reference gene) F: ATTTCCGACACGGAGAGG, 18S rRNA R: CATGGGTTTAGGATACGCTC. The amplification efficiency for each primer pair was determined from standard curves generated by serial dilutions of cDNA and was found to be between 90% and 110%. Relative expression levels were calculated using the 2^−ΔΔCt^ method and normalized to the expression of 18S rRNA. Data are presented as mean ± SD from 6 independent biological replicates (*n* = 6), with each biological replicate representing one individual fish.

### 2.6. Bacterial Strains and Direct Antimicrobial Activity Assay

The bacterial strains used in this study included *Aeromonas hydrophila* ATCC7966, *Aeromonas veronii* AV20211212, *Pseudomonas fluorescens* ATCC17386, *Escherichia coli* ATCC25922, *Staphylococcus aureus* ATCC25923, and *Streptococcus agalactiae* ATCC13813. All bacterial strains were maintained in our laboratory. *A. hydrophila*, *A. veronii*, *P. fluorescens*, and *S. agalactiae* were cultured in tryptic soy broth (TSB) at 28 °C with shaking (180 rpm) overnight and plated on tryptic soy agar (TSA) when required. *E. coli* and *S. aureus* were cultured in TSB at 37 °C with shaking (180 rpm) overnight and plated on TSA.

For antimicrobial activity assays, bacteria were collected during the logarithmic growth phase, washed three times with sterile Tris buffer (20 mM, pH 7.4), and adjusted to approximately 1 × 10^7^ CFU/mL. Equal volumes (100 μL) of bacterial suspension and different concentrations of recombinant CiCXCL14 were mixed and incubated for 6 h, yielding a final bacterial inoculum of approximately 5 × 10^6^ CFU/mL. The final concentrations of CiCXCL14 were 0.01, 0.02, 0.04, 0.08, 0.16, 0.32, 0.96 μM, respectively. Following incubation, bacterial suspensions were serially diluted and spread onto TSA plates. After overnight incubation, surviving colonies were counted, and bacterial viability was calculated based on colony-forming units (CFUs). His-tagged TrxA served as the negative control. The minimum bactericidal concentration (MBC) was determined as the lowest concentration resulting in complete bacterial killing. The EC_50_ was defined as the concentration of CiCXCL14 required to reduce bacterial survival by 50% relative to the untreated control. All experiments were conducted independently three times.

### 2.7. Time-Dependent Bactericidal Assay

The killing kinetics of recombinant CiCXCL14 were determined using a CFU assay. *E. coli* and *S. aureus* were cultured to the exponential growth phase and adjusted to approximately 5 × 10^6^ CFU/mL in Tris buffer. Bacterial suspensions were incubated with recombinant CiCXCL14 at their respective MBCs. Tris buffer alone served as the untreated control. At designated time points (0, 5, 10, 20, 40, 80, 160, and 320 min), aliquots were collected, serially diluted, and spread onto TSB agar plates. Following overnight incubation at the optimal growth temperature for each bacterial species, surviving colonies were counted, and bacterial survival was expressed as the percentage of viable bacteria relative to the untreated control.

### 2.8. Antibacterial Stability of CiCXCL14

For thermal stability analysis, CiCXCL14 was incubated at 25 °C or 100 °C for 1 h and then cooled to room temperature. To evaluate the effect of salt concentration on antibacterial activity, CiCXCL14 was diluted in buffers with different concentrations of NaCl, including 25, 50, and 100 mM NaCl, and incubated for 1 h at room temperature. After treatment, CiCXCL14 was mixed with logarithmic-phase cultures of *E. coli* and *S. aureus* adjusted to approximately 5 × 10^6^ CFU/mL. The final NaCl concentrations in the assay were 25, 50, and 100 mM, respectively. The mixtures were incubated for 6 h under optimal growth conditions. Subsequently, aliquots were serially diluted and plated onto TSB agar plates. Following overnight incubation, bacterial colonies were counted, and the residual antibacterial activity of the treated proteins was calculated relative to untreated controls.

### 2.9. Detection of Bacterial Membrane Permeability and Bacterial DNA-Binding

Changes in bacterial membrane integrity induced by CiCXCL14 were evaluated using a Propidium iodide (PI) uptake assay. *E. coli*, *S. aureus*, and *A. hydrophila* in the mid-logarithmic growth phase were harvested, washed twice with PBS, and adjusted to approximately 5 × 10^6^ CFU/mL. For the time-course experiments, recombinant CiCXCL14 was used at 0.05 μM, whereas concentration-dependent membrane permeabilization was evaluated using 0–0.1 μM recombinant CiCXCL14. PI was added to a final concentration of 5 μg/mL, and 100 μL of the bacterial suspension was transferred into black 96-well plates (Costar). Baseline fluorescence was recorded using a SpectraMax i3× microplate reader (Molecular Devices, Sunnyvale, California, USA) (excitation, 535 nm; emission, 617 nm), after which recombinant CiCXCL14 was added to the indicated final concentrations. Fluorescence was monitored every 10 min for 320 min. Bacteria treated with 0.2% Triton X-100 served as the positive control for complete membrane permeabilization. Membrane permeabilization was expressed as the percentage of PI uptake relative to the Triton X-100-treated control. Membrane permeabilization (PI uptake, %) was calculated as (Fsample − Fblank)/(Ftriton − Fblank) × 100.

The interaction between CiCXCL14 and bacterial DNA was examined by electrophoretic mobility shift assay (EMSA). Genomic DNA was extracted from *A. hydrophila* using a bacterial genomic DNA extraction kit according to the manufacturer’s instructions. A total of 400 ng genomic DNA was incubated with increasing concentrations of recombinant CiCXCL14 for 30 min at room temperature. The DNA–protein mixtures were separated on a 1% agarose gel in 1× TAE buffer at 120 V for 30 min and visualized using a nucleic acid staining system. Retardation of DNA migration was considered evidence of direct binding between CiCXCL14 and bacterial DNA.

The interaction between CiCXCL14 and bacterial DNA was further evaluated using a PicoGreen fluorescence quenching assay. Genomic DNA extracted from *A. hydrophila* (400 ng) was incubated with increasing concentrations of recombinant CiCXCL14 (0, 0.02, 0.04, 0.08, 0.16, 0.32, and 0.64 μM) in 20 mM Tris-HCl buffer (pH 7.0) at room temperature for 30 min. PicoGreen reagent (Invitrogen, USA) was then added according to the manufacturer’s instructions, and the mixtures were incubated in the dark for 10 min. Fluorescence intensity was measured using a SpectraMax i3× microplate reader with excitation at 485 nm and emission at 528 nm. The fluorescence values were normalized to those of the DNA sample incubated with an equal volume of 20 mM Tris-HCl (pH 7.0). His-tagged TrxA served as the negative protein control.

### 2.10. In Vivo Antibacterial Activity of CiCXCL14 in Grass Carp

Grass carp were randomly assigned to three experimental groups: a PBS group, an infected control group (*A. h* + Tris), and a CiCXCL14-treated group (*A. h* + CiCXCL14). Fish in the PBS group received an intraperitoneal injection of PBS only. For the survival experiment, 33 fish were included in each group (n = 33). A separate cohort of five fish per group (n = 5) was used for bacterial burden analysis. Therefore, a total of 38 fish were used for each experimental group in the in vivo study. *A. hydrophila* was cultured overnight, collected by centrifugation, washed with PBS, and adjusted to the required concentration. The challenge dose of 1 × 10^7^ CFU/fish was determined in preliminary dose-finding experiments as the minimum dose required to achieve 50% mortality within 7 days in untreated control fish, and this dose was therefore used in all subsequent challenge experiments. Fish were anesthetized with MS-222 (100 mg/L) and challenged by intraperitoneal injection with 100 μL of the bacterial suspension (1 × 10^7^ CFU/fish). At 3 h post-infection, fish in the CiCXCL14 group received recombinant CiCXCL14 (2 μg/fish in 100 μL of 20 mM Tris-HCl, pH 7.0), whereas fish in the infected Con group received an equal volume of 20 mM Tris-HCl (pH 7.0). For bacterial burden analysis, liver, spleen, and trunk kidney were collected at 72 h post-infection, weighed, homogenized in sterile PBS, serially diluted, and plated onto RS plates for colony counting after overnight incubation. Bacterial burdens were expressed as CFU/g tissue in scientific notation (×10^n^), and statistical analyses were performed using the original CFU/g values without log_10_ transformation. Survival was monitored daily for 12 days, and Kaplan–Meier survival curves were analyzed using the log-rank (Mantel–Cox) test to compare survival differences between groups. Fish exhibiting no vital signs were considered dead and recorded as mortality.

### 2.11. Statistical Analysis

All experiments were performed with at least three independent biological replicates unless otherwise stated. Data are presented as mean ± SD. Normality of data distribution was assessed using the Shapiro–Wilk test, and homogeneity of variances was evaluated using Levene’s test before parametric analysis. When both assumptions were satisfied, comparisons between two groups were performed using an unpaired two-tailed Student’s *t*-test, whereas comparisons among multiple groups were analyzed by one-way analysis of variance (ANOVA) followed by Tukey’s or Dunnett’s post hoc test, as appropriate. When the assumptions for parametric analysis were not met, the corresponding non-parametric tests were applied. A *p*-value < 0.05 was considered statistically significant.

## 3. Results

### 3.1. An Evolutionarily Conserved CXCL14 Is Present in Grass Carp

**Figure 2:**
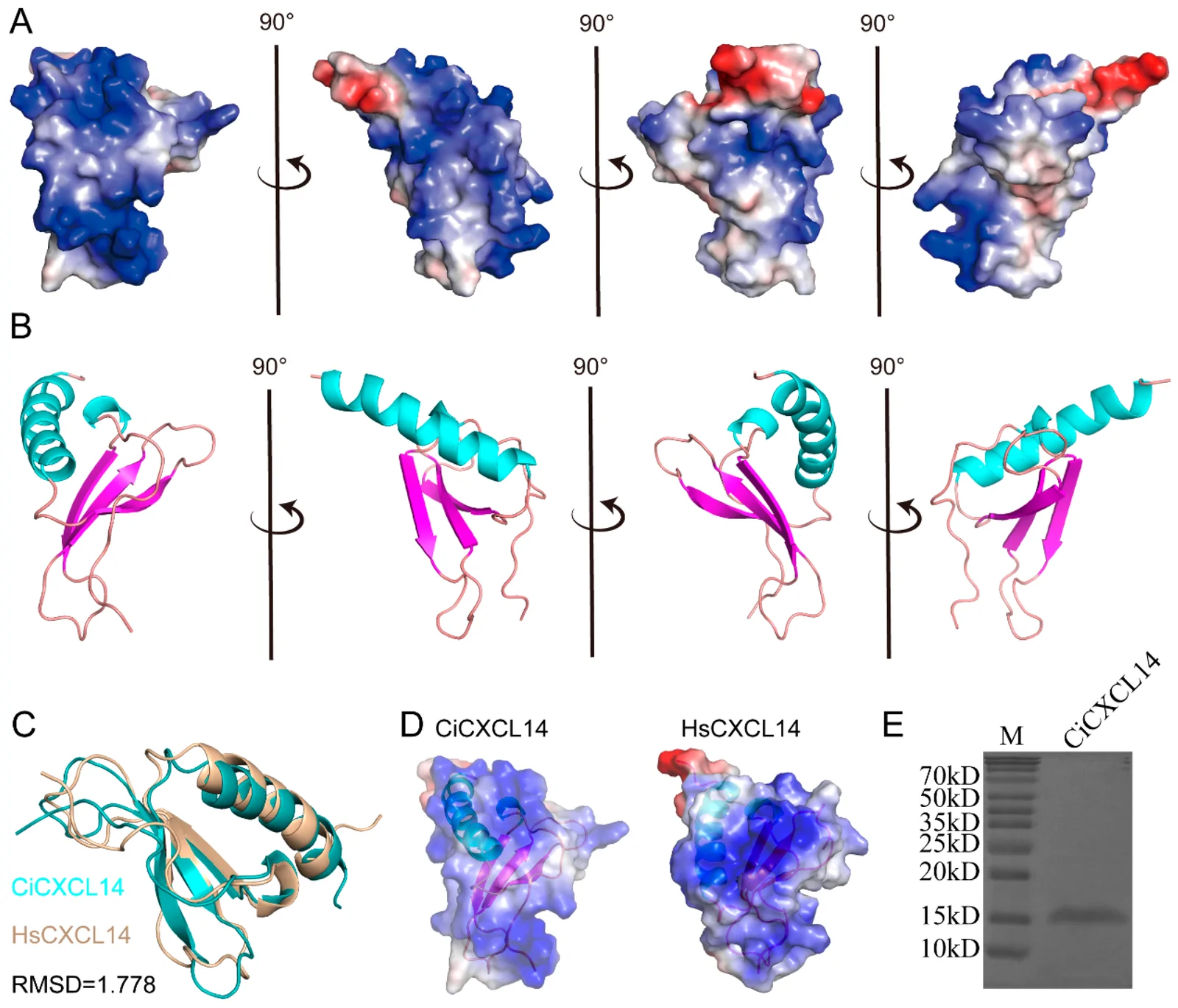
Structural modeling, electrostatic potential analysis, and prokaryotic expression of grass carp CXCL14. (**A**) Electrostatic surface potential representation of CiCXCL14. The model is rotated at 90° intervals to display the 3D distribution of surface charges. Blue, red, and white regions denote positive, negative, and hydrophobic/neutral areas, respectively. The electrostatic potential scale ranges from −87 (red, negative) to +87 (blue, positive). (**B**) Cartoon-style ribbon diagrams of the CiCXCL14 structure, corresponding to the orientations in (**A**). The α-helix is shown in cyan, β-sheets in magenta, and flexible loops in light pink. (**C**) Superimposition of the predicted tertiary structures of CiCXCL14 and HsCXCL14. The calculated root-mean-square deviation (RMSD) between the two structures is 1.778. (**D**) Comparative visualization of the surface electrostatic potentials of CiCXCL14 (left) and HsCXCL14 (right). The structures of *H. sapiens* CXCL14 and *C. idella* CXCL14 were obtained from AlphaFold predictions. (**E**) SDS-PAGE analysis confirming the prokaryotic expression of recombinant CiCXCL14. Lane M represents the protein molecular weight marker; the indicated lane shows purified intact CiCXCL14 protein. A total of 5 μg protein was loaded per lane.

The full-length coding sequence of grass carp CXCL14 (CiCXCL14) was obtained and analyzed. The open reading frame encodes a protein of 99 amino acids, which contains a putative N-terminal signal peptide of 22 residues and a mature protein region of 77 residues, as predicted by SignalP and SMART (Figure 1A). Phylogenetic analysis showed that CiCXCL14 clustered with other teleost CXCL14 orthologs, forming a distinct clade separate from mammalian counterparts (Figure 1B), consistent with the evolutionary divergence between teleost and mammalian lineages. The CXCL14 orthologs included in this analysis exhibited relatively high theoretical pI values and positive net charges (Figure 1C). Multiple sequence alignment revealed that CiCXCL14 shares 89.9% amino acid identity with zebrafish CXCL14 and approximately 58% identity with human CXCL14 (HsCXCL14), reflecting the high evolutionary conservation of this chemokine across vertebrates (Figure 1C). To gain structural insights, the three-dimensional structure of CiCXCL14 was predicted using AlphaFold3. The predicted structure revealed that cationic residues in CiCXCL14 are clustered on one surface, forming positively charged regions, whereas hydrophobic residues are predominantly distributed on the opposite surface, forming hydrophobic patches (Figure 2A). Moreover, a typical chemokine fold consisting of an N-terminal flexible region, a central antiparallel β-sheet, and a C-terminal α-helix was also observed in CiCXCL14 (Figure 2B). These structural features align with the cationic and amphipathic characteristics observed in mouse CXCL14 [11]. In addition, superimposition of the CiCXCL14 model onto the human CXCL14 structure yielded a root-mean-square deviation (RMSD) value of 1.778 Å, indicating a high degree of structural conservation (Figure 2C). However, electrostatic surface potential analysis revealed notable differences between the two orthologs. CiCXCL14 exhibited a more pronounced positively charged surface, particularly in the C-terminal region, compared with human CXCL14 (Figure 2D). This enhanced positive charge is consistent with the calculated pI values and net charges of the respective proteins (Figure 1C and Figure 2D). These findings suggest that CiCXCL14 may retain structural features associated with conserved chemokine functions in vertebrates. Recombinant CiCXCL14 was successfully expressed in *E. coli*. SDS-PAGE analysis showed a predominant band corresponding to the expected molecular mass of recombinant His-tagged CiCXCL14 (approximately 11 kDa) following Ni-NTA purification (Figure 2E). Densitometric analysis of the Coomassie-stained gel indicated that the purity of the recombinant protein was greater than 95%.

### 3.2. CiCXCL14 Is Mainly Expressed in Immune Tissues and Responds to A. hydrophila Infection

**Figure 3:**
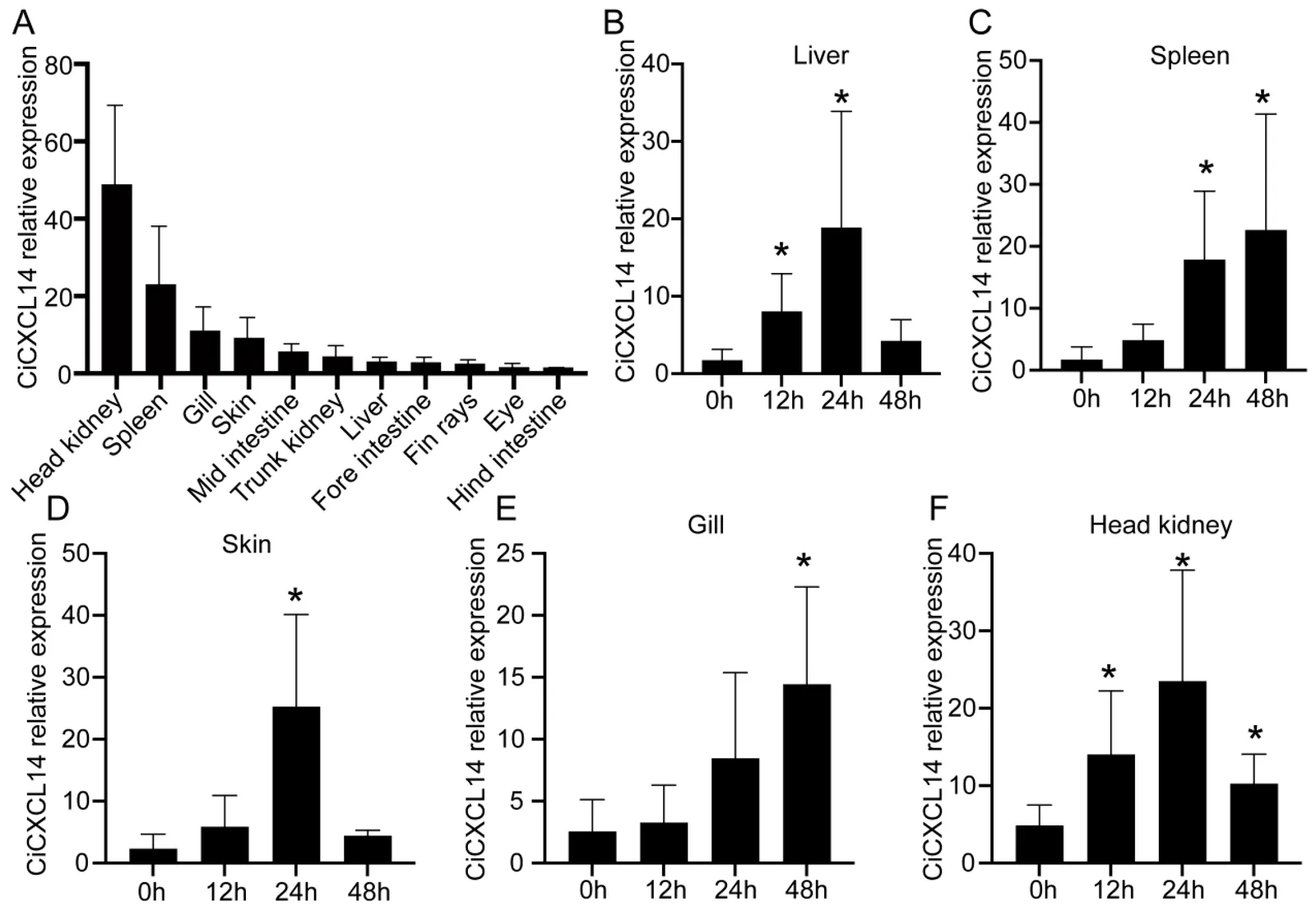
Constitutive and inducible expression of CiCXCL14 in different tissues. (**A**) The relative mRNA transcription levels of CiCXCL14 across eleven distinct tissues in healthy grass carp (n = 6 biological replicates), as determined by qPCR. (**B**–**F**) The dynamic changes in CiCXCL14 gene expression in liver (**B**), spleen (**C**), skin (**D**), gill (**E**), and head kidney (**F**) upon *A. hydrophila* challenge, as determined by qPCR. Samples were collected at 0, 12, 24, and 48 h post-infection. The vertical bars represent mean ± SD (n = 6 biological replicates). Statistical significance was determined by one-way ANOVA. * *p* ≤ 0.05.

The tissue-specific expression pattern of CiCXCL14 in healthy grass carp was first examined by qPCR analysis in eleven different tissues, including the head kidney, trunk kidney, spleen, gill, liver, skin, mid intestine, fore intestine, hind intestine, fin rays, and eye. As shown in Figure 3A, CiCXCL14 transcripts were detected in all examined tissues, with relatively high transcript levels detected in the head kidney and spleen. Moderate expression was detected in the gill and skin, while relatively lower expression was found in the mid intestine, trunk kidney, liver, fore intestine, fin rays, eye, and hind intestine (Figure 3A). This expression profile indicates that CiCXCL14 transcripts are relatively abundant in several immune-related tissues of grass carp. To investigate whether CiCXCL14 is involved in the host response to bacterial infection, we examined its expression dynamics in five key immune tissues following challenge with *A. hydrophila*. CiCXCL14 expression was significantly upregulated at 12 h post-infection, peaked at 24 h, and remained elevated at 48 h post-infection compared with the control group in the liver (Figure 3B). In the spleen, a rapid and robust induction was observed at 12 h, with expression remaining significantly elevated during the 48 h observation period (Figure 3C). In the skin (Figure 3D), which is a major mucosal barrier organ, CiCXCL14 expression increased progressively from 12 h to 24 h post-infection. In the gill (Figure 3E), a primary site of pathogen entry in fish, CiCXCL14 expression was sharply induced at 12 h and sustained at high levels through 48 h. Similarly, in the head kidney (Figure 3F), the major hematopoietic and immune organ in teleosts, CiCXCL14 expression was significantly upregulated at 12 h and remained elevated at 48 h. These results collectively demonstrate that CiCXCL14 transcripts are constitutively expressed in multiple tissues and are significantly upregulated in several immune-related tissues following bacterial challenge, suggesting a potential role in the antibacterial immune response.

### 3.3. Broad-Spectrum and Concentration-Dependent Bactericidal Activity of CiCXCL14

**Figure 4:**
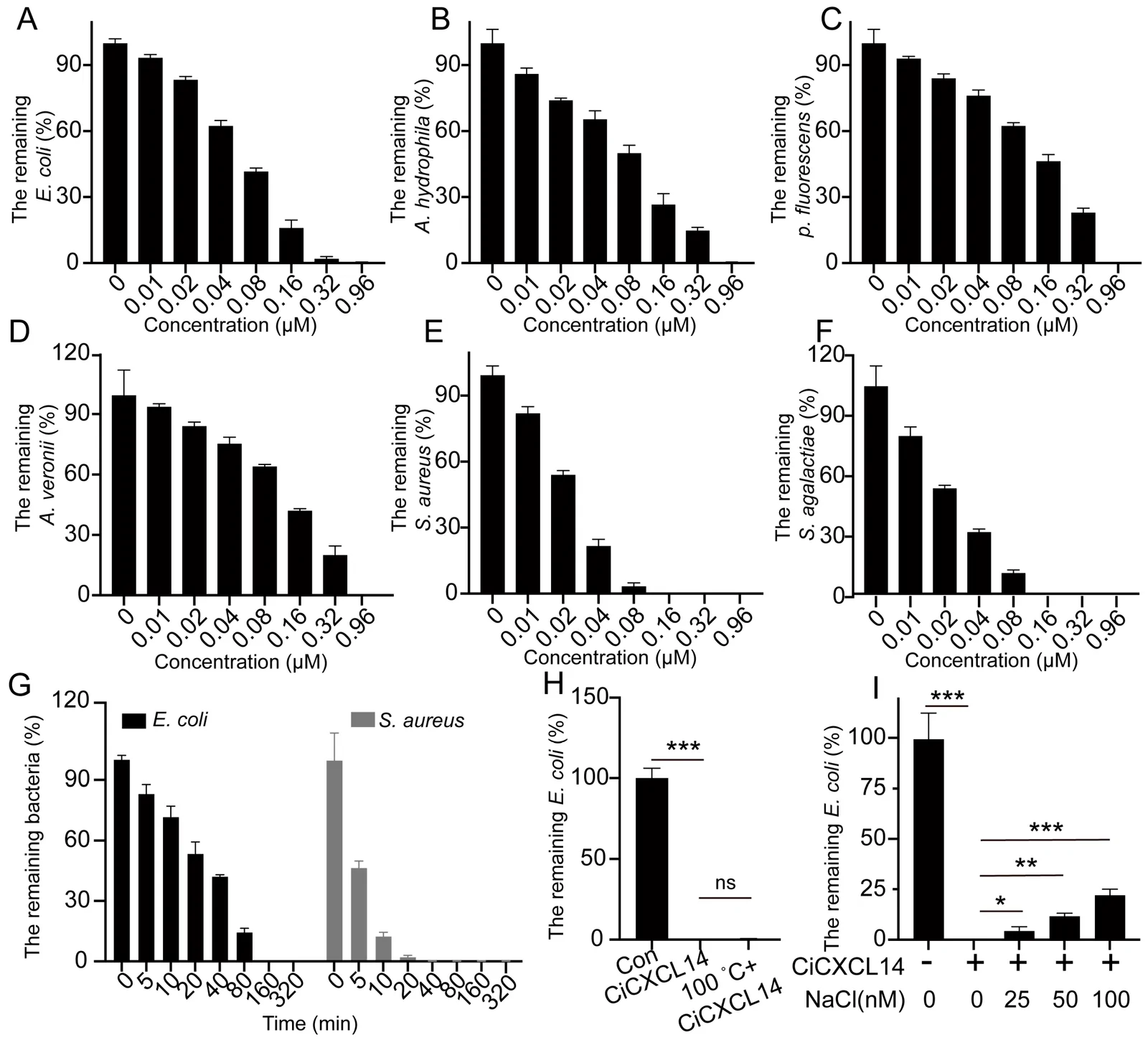
Antimicrobial characterization, stability, and ionic dependency of CiCXCL14. (**A**–**F**) Antimicrobial effects of CiCXCL14 at increasing concentrations (from 0 to 0.96 µM) on *E. coli* (**A**), *A. hydrophila* (**B**), *P. fluorescens* (**C**), *A. veronii* (**D**), *S. aureus* (**E**), and *S. agalactiae* (**F**) within 6 h. (**G**) Time-kill kinetics of CiCXCL14 against *E. coli* and *S. aureus* for different times (0–320 min). (**H**) Evaluation of structural stability. No statistically significant difference in the antibacterial activity of CiCXCL14 against *E. coli* was detected between the group subjected to heat denaturation at 100 °C for 1 h and the untreated (unheated) CiCXCL14 group. (**I**) Investigation of salt sensitivity. The antibacterial activity of CiCXCL14 against *E. coli* was measured under varying concentrations of NaCl (25 mM, 50 mM and 100 mM). “−” and “+” indicate the absence or presence of CiCXCL14, respectively. Values are shown as mean ± SD from three independent replicates. Statistical analyses were performed using an unpaired two-tailed Student’s *t*-test. * *p* ≤ 0.05, ** *p* ≤ 0.01, *** *p* ≤ 0.001, ns: *p* > 0.05.

The direct antibacterial activity of recombinant CiCXCL14 was evaluated against a panel of representative bacterial strains, including four important fish pathogens (*A. hydrophila*, *A. veronii*, *P. fluorescens*, and *S. agalactiae*) and two commonly used laboratory strains (*E. coli* and *S. aureus*). To exclude the possibility that the antibacterial activity was associated with the expression system or purification procedure, an unrelated recombinant His-tagged thioredoxin A (TrxA) protein purified using the same protocol was included as the negative control. TrxA showed no detectable antibacterial activity against the tested bacteria (Figure S1A), confirming that the observed bactericidal activity was specifically attributed to CiCXCL14. As shown in Figure 4A–F, CiCXCL14 exhibited potent, concentration-dependent bactericidal activity against all tested strains. The MBC and EC_50_ values were determined and summarized in Table S1. The MBC values ranged from 0.24 to 0.96 μM, whereas the EC_50_ values ranged from 0.09 to 0.15 μM among the tested bacterial species (Table S1), indicating that CiCXCL14 displayed strong antibacterial activity across both Gram-negative and Gram-positive bacteria. To further characterize the bactericidal kinetics of CiCXCL14, time-kill assays were performed using *E. coli* and *S. aureus* as representative Gram-negative and Gram-positive bacteria, respectively (Figure 4G). More than 50% of *E. coli* cells were killed within 40 min, and bacterial viability was almost completely abolished within 160 min (Figure 4G). *S. aureus* was more sensitive to CiCXCL14, with near-complete bacterial elimination observed within 40 min of treatment (Figure 4G). Notably, the antibacterial activity of CiCXCL14 was remarkably stable to heat treatment. Pre-incubation of CiCXCL14 at 100 °C for 1 h did not significantly diminish its bactericidal effect against *E. coli* (Figure 4H), indicating that CiCXCL14 exhibits high thermal tolerance. However, the bactericidal activity of CiCXCL14 was affected by ionic strength, as increasing NaCl concentrations (25–100 mM) progressively reduced its killing efficiency against *E. coli* (Figure 4I). Nevertheless, CiCXCL14 retained significant inhibitory activity against bacterial colony formation even in the presence of 100 mM NaCl (Figure 4I), indicating that its antibacterial activity is reduced, but not completely abolished, under elevated ionic conditions.

### 3.4. CiCXCL14 Kills Bacteria Through Membrane Permeabilization and DNA Interaction

**Figure 5:**
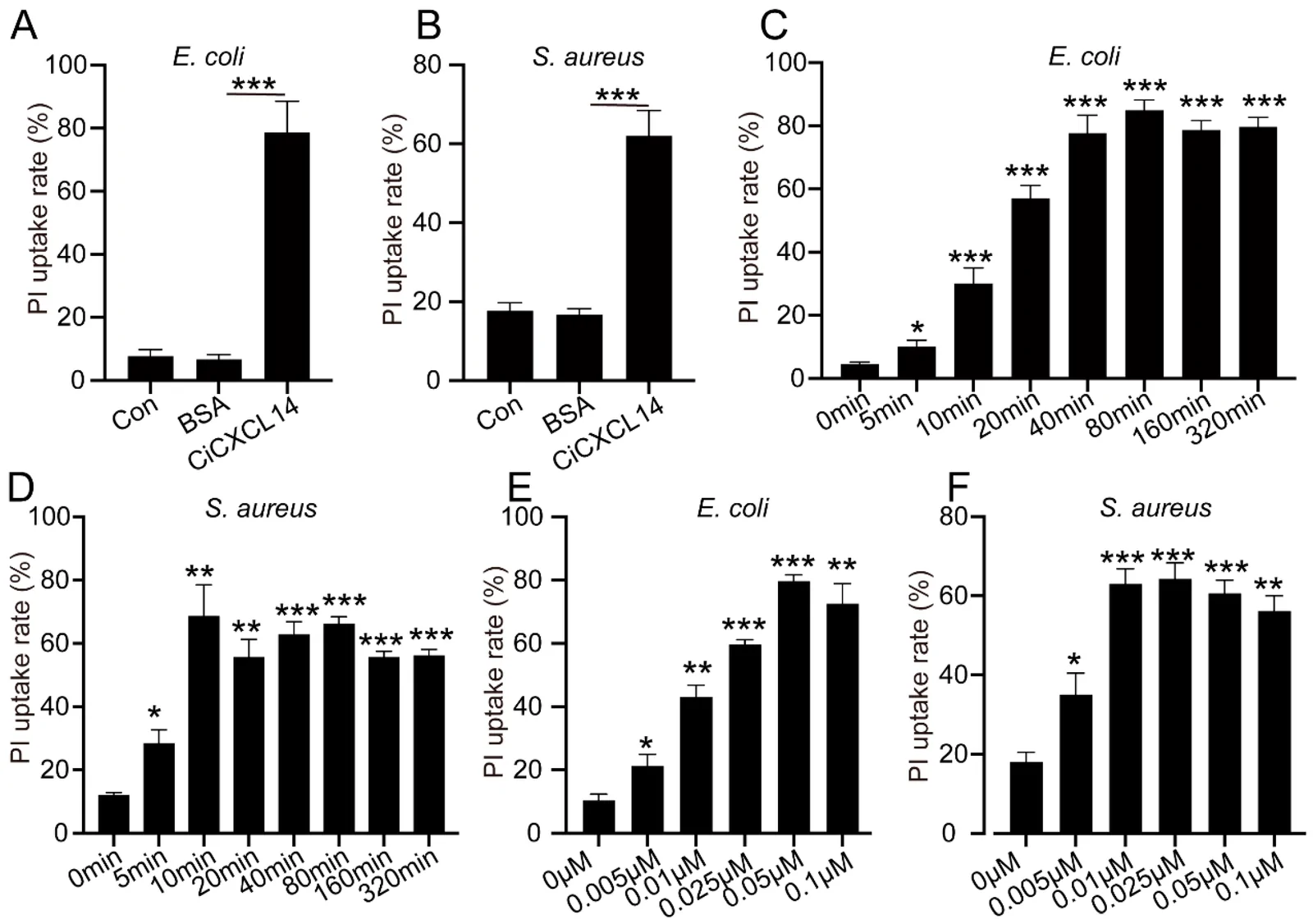
CiCXCL14 exerts bactericidal effects through disruption of the bacterial membrane. (**A**,**B**) Membrane permeabilization of *E. coli* (**A**) and *S. aureus* (**B**) following treatment with increasing concentrations of CiCXCL14, as determined by Propidium iodide (PI) fluorescence. (**C**,**D**) Time-course analysis of PI fluorescence in *E. coli* (**C**) and *S. aureus* (**D**) after treatment with CiCXCL14 (0.05 μM). (**E**,**F**) Concentration-dependent PI fluorescence induced by CiCXCL14 in *E. coli* (**E**) and *S. aureus* (**F**). Data are presented as mean ± SD from three independent biological experiments. Statistical analyses were performed using one-way ANOVA. * *p* ≤ 0.05, ** *p* ≤ 0.01, *** *p* ≤ 0.001.

To investigate whether CiCXCL14 affects bacterial membrane permeability, we examined membrane integrity using the membrane-impermeable dye PI, which enters cells with compromised membranes. Treatment of *E. coli* and *S. aureus* with CiCXCL14 resulted in a marked, concentration-dependent increase in PI fluorescence, indicating enhanced membrane permeability (Figure 5A,B). Time-course analysis showed that PI uptake was detectable within 10 min after treatment and reached a plateau by 80 min (Figure 5C,D). Consistently, higher concentrations of CiCXCL14 induced greater PI uptake over the concentration range of 0–0.1 μM (Figure 5E,F). These findings indicate that CiCXCL14 increases bacterial membrane permeability, which is consistent with membrane disruption and may contribute to its antibacterial activity.

**Figure 6:**
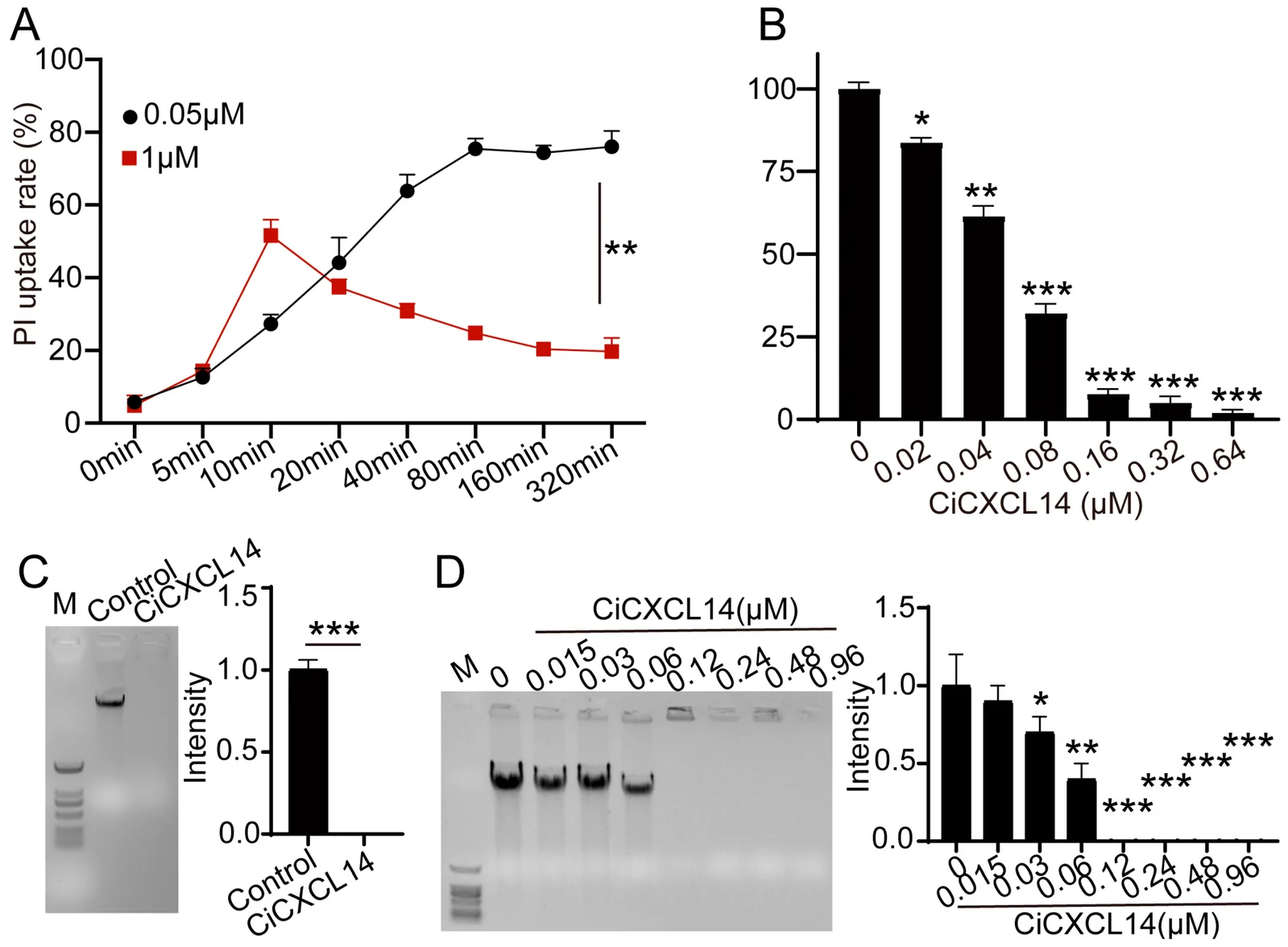
PI fluorescence kinetics at elevated CiCXCL14 concentrations and nucleic acid-binding analysis. (**A**) Time-course analysis of PI fluorescence intensity in *A. hydrophila* treated with elevated concentrations of CiCXCL14 (0.05 and 1 μM). Fluorescence was monitored at the indicated time intervals (0–320 min). (**B**) PicoGreen fluorescence assay showing the interaction between recombinant CiCXCL14 (0–0.64 μM) and 400 ng of *A. hydrophila* genomic DNA. Fluorescence intensity is expressed relative to the DNA sample incubated with an equal volume of Tris-HCl buffer (vehicle control). (**C**) EMSA showing the interaction between recombinant CiCXCL14 (0.96 μM) and 400 ng of *A. hydrophila* genomic DNA. Genomic DNA incubated with an equal volume of Tris-HCl buffer served as the vehicle control. (**D**) Different concentrations of recombinant CiCXCL14 interacted with 400 ng of *A. hydrophila* genomic DNA was showed by EMSA. Band intensities were quantified using ImageJ software and normalized to the Tris-HCl buffer-treated control. Data are presented as mean ± SD from three independent experiments. Statistical analyses for panels (**A**,**B**,**D**) were performed using one-way ANOVA. Panel (**C**) was analyzed using an unpaired two-tailed Student‘s *t*-test. * *p* ≤ 0.05, ** *p* ≤ 0.01, *** *p* ≤ 0.001.

The PI uptake assay showed that treatment with a high concentration of CiCXCL14 resulted in reduced PI fluorescence intensity, which further decreased over time (Figure 6A). To further investigate whether CiCXCL14 interacts with bacterial genomic DNA, we performed a PicoGreen fluorescence assay and an EMSA. Using a fluorescent DNA-binding assay, we found that CiCXCL14 caused a concentration-dependent reduction in PicoGreen fluorescence intensity from bacterial genomic DNA samples (Figure 6B), indicating an interaction between CiCXCL14 and bacterial genomic DNA. This interaction was further supported by gel retardation assays. Incubation of *A. hydrophila* genomic DNA with increasing concentrations of CiCXCL14 resulted in a progressive retardation of DNA migration, with the DNA band completely disappearing at higher protein concentrations (Figure 6C and D). Quantitative densitometric analysis confirmed that the DNA-binding activity was concentration-dependent (Figure 6D). Together, these in vitro biochemical assays demonstrate that CiCXCL14 is capable of interacting with purified bacterial genomic DNA. However, these assays cannot distinguish direct DNA binding from alternative effects, such as DNA aggregation, precipitation, or interference with DNA staining, nor do they establish that this interaction occurs intracellularly during bacterial killing. Therefore, the precise contribution of DNA interaction to the bactericidal activity of CiCXCL14 remains to be determined.

### 3.5. In Vivo Protective Efficacy of CiCXCL14 Against Bacterial Infection

**Figure 7:**
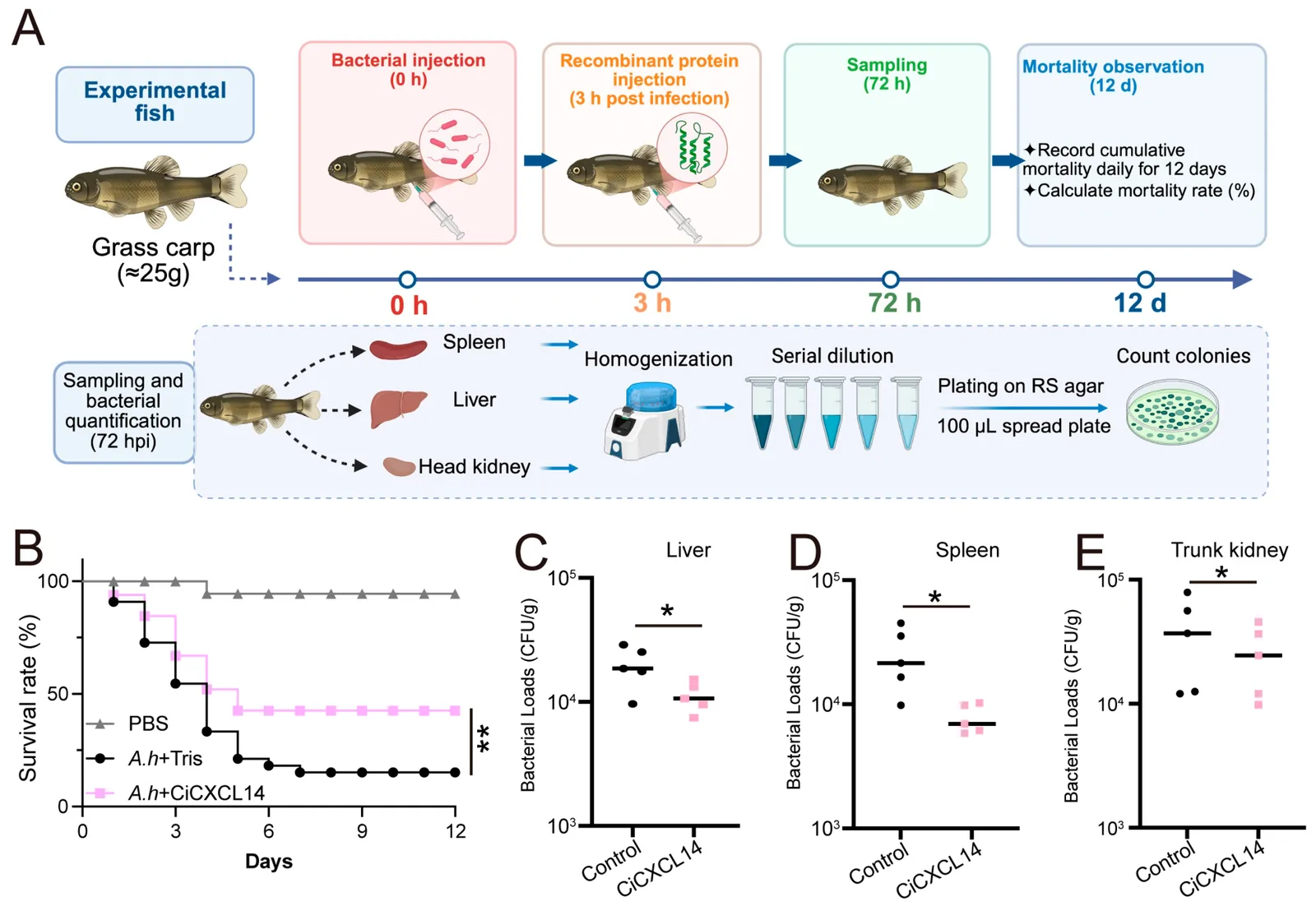
In vivo protective efficacy of CiCXCL14 against *A. hydrophila* challenge. (**A**) A diagram depicting the timeline and key procedures of the animal experiment. Grass carp were challenged with a high-mortality challenge dose of *A. hydrophila*, followed by a therapeutic intraperitoneal administration of 2 μg of CiCXCL14 or Tris-HCl at 3 h post-challenge. (**B**) The percentage of surviving animals in each group was recorded daily over a 12-day observation period (n = 33). Survival curves were analyzed using the Kaplan–Meier method and compared using the log-rank (Mantel–Cox) test. (**C**–**E**) Assessment of systemic bacterial clearance. At 72 h post-challenge, bacterial burdens (CFU/g) in the liver (**C**), spleen (**D**), and trunk kidney (**E**) were determined individually from each fish by homogenizing the tissues, serial dilution, and plating onto RS plates for colony counting (n = 5 independent fish per group). Data are presented as mean ± SD (n = 5 independent fish per group). Survival curves were analyzed using the Kaplan–Meier method and compared using the log-rank (Mantel–Cox) test. Statistical analyses for panels (**C**–**E**) were performed using one-way ANOVA based on the original CFU/g values (without log_10_ transformation). * *p* ≤ 0.05, ** *p* ≤ 0.01.

To evaluate the in vivo protective effect of CiCXCL14, grass carp were challenged with a high-mortality challenge dose of *A. hydrophila*. Three experimental groups were included: a PBS group (uninfected), an infected control group receiving Tris-HCl vehicle (Con), and an infected group treated with recombinant CiCXCL14 (2 μg/fish) at 3 h post-infection (Figure 7A). Survival was monitored daily for 12 days. As shown in Figure 7B, the CiCXCL14-treated group exhibited significantly higher survival rates compared with the Tris-HCl-treated control group. By day 12, grass carp treated with recombinant CiCXCL14 exhibited a survival rate of approximately 42%, whereas the Tris-HCl control group showed a survival rate of approximately 15%. These results indicate that recombinant CiCXCL14 improves host survival following *A. hydrophila* infection. To further assess the impact of CiCXCL14 treatment on bacterial clearance, we measured bacterial burdens in the liver, spleen, and trunk kidney at 72 h post-infection. The bacterial burden (CFU/g) was significantly lower in the CiCXCL14-treated fish compared with the Tris-HCl-treated controls across all three tissues examined (Figure 7C–E). CiCXCL14 treatment reduced bacterial burdens by approximately one order of magnitude in the liver (Figure 7C). Similarly, marked reductions in bacterial counts were observed in the spleen (Figure 7D) and trunk kidney (Figure 7E). Collectively, these findings indicate that recombinant CiCXCL14 enhances host resistance to *A. hydrophila* infection and promotes bacterial clearance in vivo.

## 4. Discussion

Chemokines have traditionally been regarded as immune signaling molecules that regulate leukocyte migration and inflammatory responses [23,24]. However, accumulating evidence over the past two decades has revealed that several chemokines possess direct antimicrobial activity and therefore function as important effector molecules of innate immunity [2,5,6,8,9,11,16]. In the present study, we demonstrated that CiCXCL14 is a direct bactericidal molecule with broad-spectrum activity against both Gram-positive and Gram-negative fish pathogens. Mechanistic analyses showed that CiCXCL14 increases bacterial membrane permeability and is capable of interacting with bacterial genomic DNA in vitro. While these findings suggest that DNA interaction may contribute to the antibacterial properties of CiCXCL14, its precise role during bacterial killing remains to be elucidated. Finally, our in vivo experiments confirmed that CXCL14 enhances host resistance to bacterial infection in grass carp. Collectively, our findings identify CiCXCL14 as a teleost chemokine with direct antibacterial activity and provide additional evidence supporting the antimicrobial potential of CXCL14 family members.

In mammals, CXCL14 is a prototypical homeostatic chemokine constitutively expressed in epithelial tissues such as the skin, gastrointestinal tract, and respiratory mucosa, with little to no expression in lymphoid tissues [25,26]. In contrast, our results show that grass carp CXCL14 is highly expressed in immune-related tissues and is markedly upregulated following bacterial infection, suggesting that its expression is dynamically regulated during the antibacterial response. Teleost fish inhabit microbe-rich aquatic environments and are continuously exposed to diverse waterborne microorganisms, placing substantial demands on their immune system [7,27]. The inducible expression of CiCXCL14 following bacterial challenge may therefore facilitate a rapid innate immune response to invading pathogens. By comparison, the constitutive epithelial expression of mammalian CXCL14 is thought to contribute primarily to immune surveillance and barrier homeostasis under steady-state conditions. These differences may reflect distinct ecological niches and host–pathogen interactions.

Previous studies have shown that mammalian CXCL14 possesses antibacterial activity against a variety of microorganisms [11]. Our results further demonstrated that CiCXCL14 exhibits broad-spectrum antibacterial activity against representative Gram-positive and Gram-negative bacteria, supporting the antimicrobial potential of CXCL14 family members. The concentration-dependent nature of the bactericidal activity we observed is consistent with the typical behavior of antimicrobial chemokines: in mammals, CXCL14 kills respiratory pathogens in a dose-dependent manner [11,25], and similar dose-dependent killing has been reported for other fish chemokines such as teleost CXCL10 and CXCL8 [9,10]. This concentration-dependent pattern indicates that higher concentrations of CiCXCL14 result in greater bactericidal activity, consistent with a dose-dependent antibacterial effect. Consistent with many cationic antimicrobial proteins, the antibacterial activity of CiCXCL14 was reduced under high-salt conditions [28,29], likely because increased ionic strength weakens electrostatic interactions between the positively charged protein and negatively charged bacterial membranes [30]. Although reduced salt tolerance may limit activity under high-ionic-strength conditions, this characteristic may be less restrictive in freshwater aquaculture. Nevertheless, improving salt tolerance through protein engineering or molecular modification may further broaden its potential applications [31].

Many AMPs, including antimicrobial chemokines CCL13, CCL28, CXCL6, CXCL9, CXCL10 and CXCL14 itself, initially interact with negatively charged bacterial membranes through electrostatic attraction, resulting in membrane permeabilization and cellular leakage [32,33,34,35]. Consistent with this model, CiCXCL14 increased bacterial membrane permeability, indicating that membrane permeabilization contributes to its antibacterial activity [32]. More intriguingly, our study demonstrated that CiCXCL14 directly interacted with bacterial genomic DNA, as evidenced by both PicoGreen fluorescence quenching and EMSA, two widely used biochemical approaches for identifying DNA-binding antimicrobial proteins [36,37]. Similar DNA-binding properties have been reported for several antimicrobial molecules, including *Micropterus salmoides* LEAP2 and LEAP2L, mudskipper β-def2 and human IL-26 [15,37,38]. In mammals, CXCL14 has also been shown to bind bacterial CpG DNA and facilitate its delivery into dendritic cells, thereby promoting TLR9-dependent innate immune activation [13,14]. Together, these findings suggest that CiCXCL14 is capable of interacting with purified bacterial genomic DNA and that DNA interaction may represent an additional mechanism associated with its antibacterial activity. However, it should be noted that the PicoGreen fluorescence assay and EMSA cannot exclude alternative explanations, including DNA aggregation, precipitation, dye displacement, or altered DNA staining. Furthermore, whether CiCXCL14 binds bacterial DNA intracellularly during bacterial killing and the precise contribution of this interaction to its bactericidal activity remain to be determined.

The in vivo protective effect of recombinant CiCXCL14 further supports its physiological relevance in antibacterial defense. Upon challenge with bacterial pathogens, administration of recombinant CXCL14 significantly reduced tissue bacterial burdens and improved host survival. Similar protective effects have been reported for CXCL12a in grass carp and CXCL14-derived peptides in mudskippers [1,16], whereas mammalian CXCL14 has been shown to promote bacterial clearance and enhance macrophage antibacterial activity [11,39,40]. In the present study, the observed protective effect was associated with reduced bacterial burdens and the direct bactericidal activity of recombinant CiCXCL14. However, whether CiCXCL14 exerts similar immunomodulatory effects in grass carp remains to be determined.

From an application perspective, the broad-spectrum antibacterial activity and in vivo efficacy of recombinant CiCXCL14 suggest its potential as a candidate antimicrobial molecule for aquaculture. Given the increasing challenges posed by bacterial diseases and antimicrobial resistance, host-derived antimicrobial molecules have attracted growing interest as potential alternatives to conventional antibiotics [41]. However, the present study did not evaluate the toxicity, immunogenicity, pharmacokinetics, environmental stability, or long-term safety of recombinant CiCXCL14. Therefore, further studies addressing these aspects, together with dose optimization and validation under aquaculture conditions, will be required before practical application can be considered.

## 5. Conclusions

This study identifies CiCXCL14 as a teleost chemokine with direct antibacterial activity and demonstrates that it is highly expressed in immune-related tissues and is upregulated following bacterial infection in grass carp. CiCXCL14 exhibited broad-spectrum, concentration-dependent bactericidal activity and was associated with increased bacterial membrane permeability and interaction with bacterial genomic DNA in vitro. In addition, administration of recombinant CiCXCL14 reduced tissue bacterial burdens and improved survival following *A. hydrophila* infection. Collectively, these findings expand our understanding of the antibacterial functions of teleost chemokines and provide a foundation for future studies investigating the biological functions and potential applications of CiCXCL14 in aquaculture.

## Data Availability

Data will be made available on request.

## Supplementary Materials

The following supporting information can be downloaded at: https://www.mdpi.com/article/10.3390/ani16162458/s1, Figure S1: The bactericidal and nucleic acid binding properties of CiCXCL14. (**A**) Antimicrobial effects of CiCXCL14 or TrxA at 0.5 µM on *E. coli* within 5 h. (**B**) Fluorimetric quantification of *A. hydrophila* DNA staining by picogreen dye after mixing of *A. hydrophila* DNA with CiCXCL14 or TrxA at 0.1 µM.; Table S1: MBC and EC50 determination of CiCXCL14 antimicrobial activity against several microorganisms.
