# Supplementary material for: CiCXCL14 Exhibits Broad-Spectrum Bactericidal Activity and Protects Grass Carp Against Aeromonas hydrophila Infection
Source: Animals (Basel). 2026 Aug 7;16(16):2458. doi: 10.3390/ani16162458 (PMC13508940; doi:10.3390/ani16162458)
Supplement: Supplementary file 1 [file animals-16-02458-s001.zip › animals-4428952-supplementary.pdf]

CiCXCL14 Exhibits Broad-Spectrum Bactericidal Activity and Protects Grass Carp Against *Aeromonas hydrophila* Infection

Ya-Zhen Hu [1], Yu Chen [2], Weicheng Wang [2], Bo Yuan 2, Youfeng Xie [2], He Zhao [3]

Huijie Chen [2], Wentao Zhu [2*]

[1]*State Key Laboratory for Quality and Safety of Agro-Products, Ningbo University, Ningbo 315211, China;*

[2] *College of Life Science and Technology, Tarim University, Alar 843300, China*

3 International Joint Research Center for Coral Reef Ecology of Hainan Province, School of Ecology, Hainan University, Haikou 570228, China

Keywords: CXCL14; Antimicrobial protein; DNA binding; Membrane disruption

*Corresponding author (Wentao Zhu, email: zhuwt@mail.taru.edu.cn)

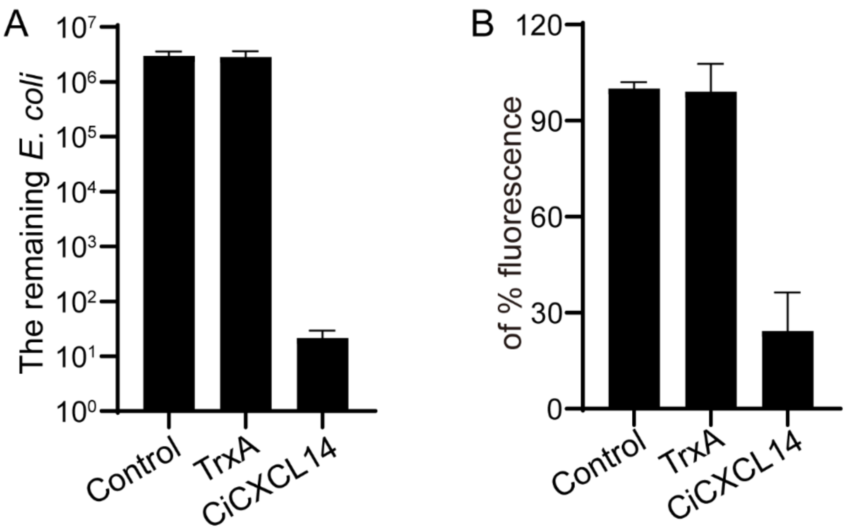

Figure S1. The bactericidal and nucleic acid binding properties of CiCXCL14. (**A**) Antimicrobial effects of CiCXCL14 or TrxA at 0.5 μM on *E. coli* within 5 h. (**B**) Fluorimetric quantification of *A. hydrophila* DNA staining by picogreen dye after mixing of *A. hydrophila* DNA with CiCXCL14 or TrxA at 0.1 μM.

Table S1. MBC and EC50 determination of CiCXCL14 antimicrobial activity against several microorganisms.

Bacterial Strain	MBC	EC50 (MBC50)
Aeromonas hydrophila (ATCC 7966)	0.96 μM	0.15 μM
Aeromonas veronii (ATCC 35622)	0.96 μM	0.14 μM
Pseudomonas fluorescens (ATCC 17397)	0.48 μM	0.13 μM
Escherichia coli (ATCC 25922)	0.96 μM	0.12 μM
Staphylococcus aureus (ATCC 25923)	0.24 μM	0.09 μM
Streptococcus agalactiae (ATCC 13813)	0.48 μM	0.11 μM
